# Can adults automatically process and translate between numerical representations?

**DOI:** 10.1098/rsos.250856

**Published:** 2025-07-16

**Authors:** Iro Xenidou-Dervou, Caroline Appleton, Serena Rossi, Natasha Guy, Camilla Gilmore

**Affiliations:** ^1^ Centre for Mathematical Cognition, Department of Mathematics Education, Loughborough University, Loughborough, UK

**Keywords:** numerical cognition, working memory, numerical representations, dual-task paradigm, cross-modal comparison

## Abstract

Arithmetic and the ability to use numbers are important skills. Numbers can be represented in three ways: through number words, Arabic symbols or non-symbolically. Much research attention has focused on how associations form between these three numerical representations. However, it is not yet clear whether these associations are automatic or if they require working memory (WM) resources. In this registered report, we used the dual-task paradigm to answer this question. Eighty-one adults were administered dot, digit and cross-modal (i.e. dot versus digit) magnitude comparison tasks in standalone and dual-task conditions with phonological (PL) or visuospatial (VSSP) WM interference. We found that all three types of magnitude comparison necessitated WM resources. Symbolic comparison necessitated VSSP WM. Surprisingly, in this task, accuracy improved under both WM interference conditions, evidencing the proposal that introducing executive function challenges in simple and familiar tasks can improve performance. In non-symbolic comparison, our findings demonstrated that the VSSP and the PL—albeit to a lesser extent—were employed. Finally, cross-modal comparison necessitated VSSP WM. These findings evidence the fundamental role visuospatial processing plays in numerical processing and that adults require WM resources for simply processing numerical representations and translating between them.

## 1. Introduction

Basic arithmetic skills, including counting and learning Arabic symbols, are acquired early in childhood. However, they have far-reaching consequences, including predicting future arithmetic skills, wider educational achievement [1] and future socioeconomic status [2]. Arithmetic is a skill that is important for everyday life, for example, for telling the time and buying food, and yet 25% of adults in the UK do not have the required numeracy skills for such day-to-day tasks [3]. In our daily lives, we encounter numbers in different forms; number words (the word ‘five’), symbolic (the Arabic symbol ‘5’) or non-symbolic (5 apples). Despite the importance of numbers and arithmetic skills in every aspect of life, it is not yet clear how we process numerical representations. The present study aimed to determine how far working memory (WM) and its components are involved in processing numerical representations.

### 1.1. The nature of numerical representations

Much research attention has been focused on how we represent numbers and how the nature of these representations is related to arithmetic both in children and adults (e.g. [4–6]). Here we use ‘representation’ to mean internal representations, how numbers are represented cognitively and how these representations are linked together to provide meaning, rather than external physical representations of numbers. Numerical information can be represented in three ways: through words (often verbally), in a visual Arabic number form or non-symbolically [7].

Number words are the first exact symbolic representation to be learnt in childhood. Children begin to recite the count sequence around their second birthday and by their third birthday begin to attach meaning to single-digit number words [8]. Knowledge of the verbal count sequence is associated with success in later numeracy [9] and is an important stepping stone to future arithmetic skills.

Like number words, Arabic symbols allow exact representations of quantities [10]. They are a powerful tool, which allows us to concisely represent, access and manipulate exact numbers. Understanding of Arabic symbols is associated with arithmetic skills, both in children (e.g. [11,12]) and in adults [13]. This stands to reason, as Arabic symbols are required to access the arithmetic curriculum in schools and to understand most numerical information presented to us. Furthermore, Arabic symbols encapsulate other mathematical constructs, such as place value, which are important for wider mathematical understanding [14].

A third way that numbers can be represented is non-symbolically and research indicates there are two systems for this: one for small, exact numbers and one for large, approximate numbers. The small, exact system is known under various names across research, for example, the object tracking system [15] and is often associated with subitizing [16]. The subitizing range refers to the quantities which can be quickly and exactly enumerated, up to three in children and four in adults [17]. This system has been evidenced by research which finds that when enumerating a set of objects, accuracy decreases and reaction times increase significantly when the quantity increases above four [18].

The system for processing large numbers has been referred to as analogue magnitudes or the approximate number system (ANS) (e.g. [7,19,20]). The ANS is assumed to provide estimates of the quantity that a given set of non-symbolic stimuli represents. Repeated presentations of the same quantity result in slightly varying estimates; hence, mental representations of quantities are approximate in the ANS [21]. The precision of one’s ANS can be described as the reliability of activated estimates around the true quantity [22]. In research, the ANS is commonly measured using dot comparison tasks; participants are presented with two dot arrays and asked to select which is the larger (e.g. [23]). Correlational and experimental evidence in children and adults suggest that widely used ANS tasks require domain-general capacities, such as WM and inhibition skills [24–27].

It has been suggested that the precision with which individuals can represent and process non-symbolic quantities is associated with success in arithmetic. For example, Libertus *et al*. [28] found that accuracy on a dot comparison task related to later arithmetic ability. However, the evidence for the relationship between the ANS and arithmetic is mixed [29]. Some research suggests that factors other than the numerosity of a set may influence the relationship with arithmetic [24], and other research suggests that there may be mediating factors, such as symbolic number knowledge [30,31].

Given that there are three representations of number, it raises the question of how these representations are connected, and whether it is in fact the connections between these representations that are more important for arithmetic, rather than the representations themselves. We now turn to a discussion of the research thus far into the connections between representations; here, we present the evidence in the order that connections are thought to form in children [32].

### 1.2. Translating between numerical representations

Dehaene [7] proposed the triple-code model as a way of explaining how numerical representations are related. The triple-code model describes the way numbers may be represented mentally in three different ‘codes’, what we refer to here as internal representations. As described above, numbers can be represented with number words (e.g. ‘three’), through Arabic symbols (3), or through an analogue magnitude code, which is a representation of quantity [7]. These three representations of numbers can be linked together, allowing input in one representation and output in another. Throughout this study, we will use the phrase ‘translation’ to describe the links between the different representations of a quantity. There is evidence from a range of sources that translating between numerical representations (i.e. intentionally converting or comparing quantities in different representations) is important for arithmetic abilities.

#### 1.2.1. Translating between number words and non-symbolic quantities

Translating between number words and quantity representations has been well studied in small numbers. This association is often referred to as cardinality, the principle that non-symbolic quantities can be represented by symbolic quantities [33]. Young children are thought to gain this understanding around their fourth birthday [34,35]. Hutchison *et al*. [36] propose that because small quantities are processed exactly, for example through the object tracking system [19], they are processed more similarly to symbolic representations (either Arabic or number words) than to large quantities processed through the ANS. This may explain why forming associations between symbolic (Arabic or number words) and non-symbolic representations in small numbers is easier than in large numbers.

However, both children [37] and adults [38,39] struggle with translating non-symbolic quantities to number words in quantities outside the subitizing range. It is suggested that translating from a large approximate non-symbolic quantity (processed via the ANS) to an exact number word is cumbersome and may cause difficulties [39]. The ability to form these associations is related to arithmetic [37] and therefore being able to represent these inaccurate non-symbolic quantities with a number word appears to be important. This highlights why we must consider the size of quantities (i.e. within or beyond the subitizing range) when examining the nature of numerical representations.

#### 1.2.2. Translating between Arabic symbols and number words

Translating between digits and number words has also been found to be related to arithmetic [40]. Being able to provide a number word for an Arabic symbol was found to be related to later formal arithmetic achievement in kindergarten children [11]. Similarly, digit naming was the only factor which predicted growth in arithmetic in primary school children across a 2-year period [41], and these findings were replicated by Habermann *et al*. [42]. Number words and symbolic representations are both exact representations of numbers [10] and therefore accuracy in these tasks is often higher than in translations involving non-symbolic quantities.

#### 1.2.3. Translating between Arabic symbols and non-symbolic quantities

Less research has focused on translating between Arabic symbols and non-symbolic quantities; however, these associations are also related to arithmetic abilities. For example, Brankaer *et al.* [4] found that children who were more accurate at matching dot arrays (non-symbolic quantities) to their Arabic symbols had higher arithmetic achievement.

These associations are often measured using cross-notation or cross-modal comparison tasks, where participants are presented with a symbol and a dot array and asked to select the larger. As with translating between non-symbolic quantities and number words, there is evidence that adults are particularly poor at these tasks [38]. Izard & Dehaene [43] found that when asking participants to estimate the numerosity of a dot array, participants significantly underestimated the true quantity. Furthermore, Lyons *et al.* [44] found that reaction times were significantly higher when completing cross-notation tasks (translating between Arabic symbols and non-symbolic quantities) than when completing dot and digit comparisons (processing of non-symbolic quantities or Arabic symbols). These findings suggest that there may not be a direct association between Arabic symbols and non-symbolic quantities. In particular, we do not yet know whether adults can directly translate between Arabic symbols and non-symbolic quantities or whether access to number words is necessary for this process.

At present, research into translations between the different representations of numbers has not made the distinction between the two non-symbolic processing systems, the ANS and the object tracking system. For reasons highlighted above, primarily the differences between the small, exact system and the approximate large system, it is important to examine the nature of numerical representations in quantities of different sizes (i.e. within and beyond the subitizing range) separately.

### 1.3. The nature of translations between number representations

The aforementioned literature establishes the importance of forming strong associations between the different forms of number representations for adults’ arithmetic skills, but little is known about the nature of these associations in adulthood. Several models attempt to explain the relationship between representations [45] and particularly how representations come to gain meaning. In the triple-code model, as described above, semantic meaning of words and symbols is only provided through the connection with the non-symbolic quantity [7]; this suggests that translations between representations are activated automatically to provide meaning.

The studies above focused on tasks where individuals intentionally translate or compare representations. Other research suggests that we may automatically translate one type of representation to another, even where it is not necessary for the task being undertaken. Studies have examined the automaticity of number processing using several methods. Reynvoet & Brysbaert [46] used a priming study to investigate the automaticity of translations between Arabic and verbal representations. Participants were presented with either an Arabic digit or verbal number word (the prime) and then the alternative representation (the target) and asked to specify whether the target was odd or even. Where the prime and the target were numerically closer, response times for the parity judgement task were lower, suggesting that participants were automatically processing the numbers in their different modalities.

Automaticity of number processing has also been measured using congruency studies [47]. In these studies, participants are asked to judge which is the physically larger of two Arabic digits, while ignoring numerical size. Where the numerically larger digit is also physically larger, the trials are congruent and reaction times are lower. However, where trials are incongruent, reaction times are higher [48]. From this, it is inferred that participants are automatically accessing the non-symbolic quantity of the Arabic digit.

Furthermore, number words have been found to influence the processing of Arabic symbols in both adults and children, as seen in inversion effects, demonstrated in languages such as Dutch and German where number words are inverted [49–51]. This shows that representations of numbers in one modality can be influenced by a different modality, and that the processing of these representations may be automatic, i.e. that verbal representations are automatically activated when processing Arabic symbols, even where number words are not necessary (or relevant) to the task.

Neuroscientific studies have provided further evidence of the automaticity of letter and number processing. The processing of letters can be thought of in a similar way to number processing; both involve the association between a visual form (the letter shape or Arabic symbol) and a verbal sound (the letter or number sound). A neuroimaging study found that when congruent letters or numbers (i.e. the matching symbol and sound) were presented, patterns of brain activation were similar and higher than when non-congruent pairs were presented [52]. Notably, the ability to form these automatic representations between letter–sound pairs has been found to relate to reading ability [53].

### 1.4. The role of working memory in numerical processing and translation

The studies described above considered automaticity in terms of the involvement of different numerical representations in tasks where they were not necessary. An alternative approach to automaticity is to consider the involvement of WM; where skills are automatized, there is thought to be no WM involvement [54].

WM is a cognitive system where information is held and manipulated in the mind [55]. A commonly used theoretical model of WM is Baddeley and Hitch’s, a multi-component, limited-capacity system designed for storing and processing information [56]. It is thought to consist of the visuospatial sketchpad (VSSP) and the phonological loop (PL), which are responsible for storing information in the respective modalities [57], and the central executive (CE), which is responsible for processing information and regulates, controls and monitors the subsystems. It also contains the episodic buffer, which is responsible for combining information from the slave systems and from long-term memory [58]. At present, the role of the episodic buffer in numerical cognition is not well understood and is not the focus of the present study.

Correlational studies can provide indirect evidence about the role of WM in processing numbers in children [27,31,59]. Across multiple studies in school-aged children, the PL has been found to relate to symbolic abilities, including tasks such as counting, digit naming and symbolic comparison tasks [60–62]. Purpura & Ganley [61] also found that the PL related to a non-symbolic comparison measure, while Yang *et al*. [62] found the VSSP to be related to the ANS. These mixed findings provide evidence that WM is related to representing numbers and quantities. The above research is all in children. To the best of our knowledge, no correlational research has examined the role of WM in numerical processing in adults. Furthermore, correlational studies cannot tell us whether WM resources are required for processing (i.e. comparing or manipulating numerical representations within a particular code: verbal, symbolic, non-symbolic) and translating (i.e. converting or comparing numerical representations across codes) numerical information, only that they are related.

Using the theory of WM, it is possible to use an experimental design to examine if certain components are *required* for the processing of numerical information. In studies using the dual-task paradigm participants complete a primary task (the task of interest, which is assumed to involve some aspect of WM), alongside a secondary, interference task known to involve a component of WM. If the primary task requires the component of WM to be interfered with or suppressed by the secondary task, then performance on either the primary or secondary task will break down in comparison to control standalone conditions, i.e. without a dual-task load (for a review, see [63]). Such an experimental design can be evidence of the causal role of WM in processing numerical information.

Few studies have so far used the dual-task paradigm to determine the role of WM in adults’ symbolic number processing [64–66]. In Maloney *et al*. [65], adult participants completed a single-digit Arabic comparison task under two conditions, no load and phonological load. In the phonological load condition, participants were presented with a letter span before the comparison task, and then asked to recall the span after each comparison trial. Results showed that under the phonological load, performance in the symbolic comparison task was impaired in contrast to the no load condition, suggesting that the phonological loop is required in the processing of Arabic symbols. However, by only using a phonological secondary task, it is not possible to tell whether the effects found were due to the phonological interference specifically, or due to the increased cognitive load of completing two tasks simultaneously. Van Dijk *et al.* [66] and Herrera *et al*. [64], on the other hand, imposed both verbal and visuospatial WM load on adults’ symbolic magnitude comparison processing. In these studies, symbolic comparison was assessed with a task where participants saw an Arabic digit ranging from 1 to 9 and must indicate whether the number they saw is smaller or larger than 5. Performance in this type of task elicits the so-called spatial numerical association of codes (SNARC) effect [67], which reflects an association between numerical magnitude and response side, such that larger numbers are associated with the right side and smaller with the left. In both studies, under the spatial—but not the verbal—load the expected SNARC effect was not observed. These findings demonstrate that the VSSP may play a role when processing the spatial representation of numbers. Given the key differences in the primary task used across these studies, the question remains: Which component of WM is necessary when processing and translating between different number representations?

### 1.5. The present study

The present study investigated the processing of Arabic symbols and non-symbolic quantities, and the role of verbal representations in translating between symbolic and non-symbolic representations. Using a robust, dual-task design, we can determine which WM components are involved in the processing and translation of numerical representations. If associations between representations are processed automatically, then we would expect to see no WM involvement.

To examine the processing of numerical representations, we administered dot comparison, digit comparison and cross-modal comparison tasks as primary tasks, which were conducted in standalone and dual-task (phonological and visuospatial) conditions. This allowed us to compare performance under PL and VSSP interference, ensuring that any detriment observed in task performance is due to the targeted WM component interference.

Examining performance across all three primary tasks allowed us to draw conclusions about the specific nature of numerical representations when both processing and translating different representations. The use of three comparison tasks allowed us to draw conclusions about the nature of each representation and ensured that any WM involvement would be due to the specific representation and not to the act of comparing any two quantities. If performance on the cross-modal task is impacted by the dual-task conditions but performances on the digit comparison and dot comparison tasks are not, then we know that WM is required for the process of translation, and not for simply processing the numerical representations themselves.

This method allowed us to answer further questions about the nature of representations in each modality. We expected to see phonological involvement in the symbolic comparison task; however, previous research is less clear about the WM involvement in dot comparison tasks, and, therefore, we aimed to clarify this. Maloney and colleagues [65] found phonological involvement in a cross-modal mapping task; however, they did not investigate VSSP involvement.

As discussed, non-symbolic numbers are processed through two different systems, the ANS for large numbers and the small exact system for small numbers. Therefore, to fully understand the translation of non-symbolic quantities to number words and Arabic representations, we must consider both non-symbolic representational systems. The present study, therefore, also examined the differences in how small (1–4) and larger (5–9) quantities are processed and translated. We chose these quantities, rather than quantities greater than 10, as whilst the non-symbolic representations are approximate, it is still possible for adults to attach Arabic symbols to these quantities. We expected that quantities in the small range would involve more phonological processes than those in the large range because small non-symbolic representations are assumed to be processed in a similar way to symbolic representations [36].

To address our primary research question, we designed secondary tasks that could interfere with the PL or VSSP components of WM. We aimed to address the following research questions:

(1) Are symbols and non-symbolic representations accessed automatically or does access require the involvement of WM components?
  (a) We hypothesized that the processing of Arabic digits will require the involvement of the PL.
  (b) We hypothesized processing of non-symbolic quantities will require the involvement of the VSSP.
(2) Can adults translate between symbolic and non-symbolic representations automatically or does translation require access to verbal representations?
  (a) We hypothesized that translation between symbolic and non-symbolic representations will require access to the PL.
(3) Does the processing of numerical information differ for small and large quantities?
  (a) We hypothesized that for symbolic processing and cross-modal translation, there would be no differences between small and large quantities and that both would require access to the PL.
  (b) We hypothesized that for non-symbolic quantities, small quantities will be processed automatically and large quantities will be processed using the VSSP.

The Stage 1 manuscript (https://osf.io/z9cv2) and related resources (e.g. stimuli, experiment scripts) for the following experiment can be found at: https://osf.io/ktq8e/.

## 2. Method

### 2.1. Participants

Eighty-one adult participants (*M*_age_ = 25.32; s.d. = 11.09) took part in the experiment (*n* = 50 identified as women, *n* = 30 as men and *n* = 1 responded ‘other’). Adult participants (age 18−65) were recruited via university email and social media (*N*_students_ = 63, *N*_other_ = 18). Research has shown that there is relatively little change in adults’ WM performance within this age range [68]. Participants had normal or corrected-to-normal vision and hearing and spoke English as their first language. Ethical approval was granted by Loughborough University Ethics Committee and participants were reimbursed for their time.

### 2.2. Power analyses

We conducted *a priori* power analyses to calculate the required sample size using G*Power [69,70]. Prevailing theories of number processing such as the ANS and triple code model have been developed for explaining individual differences in *accuracy*. Therefore, we based our power analysis on our assumptions of the minimum effect size of interest in accuracy [70].

All power analyses were calculated using an alpha level of 0.05 and a minimum power of 90%. For primary tasks, there were a total of 160 trials. We calculated our minimum effect size of interest by considering what we believe to be the smallest relevant decrease in performance. Previous studies have demonstrated that adults’ accuracy rate on standalone comparison tasks of this type is very high (e.g. dot comparison accuracy: 99.7%, s.d. = 0.3 in Lyons *et al*. [44]). Given the expected high performance, we decided to power our study so that we could detect a difference of 5 out of 160 trials on the primary task; this would reflect a 3% difference, which we believe would be a meaningful decrease in performance. Based on these calculations, the largest required sample size was *n* = 81, and therefore this was the sample size that was recruited for this experiment. For RT, this would allow us to detect differences of 50 ms for the symbolic and non-symbolic comparison conditions and a difference of 80 ms for the cross-modal comparison condition. For secondary tasks, there was a total of 20 trials. A decrease in 1 sequence length (e.g. remembering 6 items versus 5 items) would mean a decrease of 4 out of the 20 trials of the secondary task. We expect adults to remember on average up to 6 items [71]; therefore, we expected the mean for the PL secondary task to be 16 (the number of correct sequences out of 20 if one correctly recalls 6 items). We decided that a drop of 4 out of 20 trials (i.e. 1 sequence length) would be a meaningful decrease in performance for our experimental design. Although calculations of our smallest effect sizes of interest were informed by our theoretical predictions and practical considerations, they could also inevitably be considered arbitrary since no study has previously examined these effects, therefore non-significant results should be treated tentatively. Calculations of effect sizes can be found in appendix A, outputs for the largest power analysis can be found in appendix B, and all other outputs can be found in the dedicated Open Science Framework (OSF) directory: https://bit.ly/3lFeWll.

### 2.3. Materials

#### 2.3.1. Primary tasks

##### Numerical comparison tasks

Participants completed symbolic, non-symbolic and cross-modal comparison tasks. The quantities used in each task were the same. Small numbers comprised 1−4, and large numbers 5, 7 and 9. These numbers were selected to ensure that the ratios between the numbers were large enough for participants to make judgements about which is larger using non-symbolic representations, and to equate the ratios across the small and large numbers. All unique combinations of these number pairs within sizes (small exact versus ANS) were used, with the exception of pairs with a ratio of 0.25, which were removed in order to equate difficulty across the small and large sets. Eleven was added to the large set, to ensure that participants did not always select 9 as the larger quantity; however, these trials were excluded from the analysis. Further details about the quantities can be found on OSF. In the cross-modal comparison task, the side of presentation for the Arabic symbol was counterbalanced.

Quantities were presented on the screen, and participants were instructed to select the larger quantity and respond using the keyboard (‘z’ if the left quantity was larger, ‘m’ if the right quantity was larger). Quantities appeared on the screen for 1000 ms, to prevent counting; however, participants could respond indefinitely. Dot arrays were created using MatLab and we controlled for visual properties such as surface area. Comparison pairs were created such that across all trials, no one property of the arrays (diameter, surface area, convex hull, density or contour length) would allow 100% accuracy. In half of the trials, visual parameters were congruent with quantity (i.e. the array with the higher quantity of dots also had larger diameter, greater density, etc.), and in half of the trials, visual parameters were incongruent with dot quantity [72].

### 2.3.2. Secondary tasks

#### Phonological

A reverse letter span task was used as a secondary task to load the PL component of WM. The sequence of events was as follows. (i) Participants were presented with a randomized sequence of letters (1 s per letter, presented orally through the computer) and told to remember the sequence. Each letter could only appear once in a sequence. Letters were chosen from the set ‘F, H, J, K, L, N, P, Q, R, S, T, Y’, as used in Maloney *et al*. [65]. (ii) After completing eight trials of the primary task (approx. 8 s), participants were then asked to recall the sequence in reverse, with the response being entered into the computer by the experimenter. By recalling the sequence in reverse, it required participants to use their WM to process the information, as opposed to simply maintaining the letters in short-term memory.

The span ranged from three to seven letters, increasing in length throughout each condition, as this was found to be the range that an average adult can remember in a standalone reverse span task [71]. Four trials were used for each span length, resulting in a total of 20 trials. For the secondary task, we recorded the accuracy of recall.

#### Visuospatial

**Figure 1.**
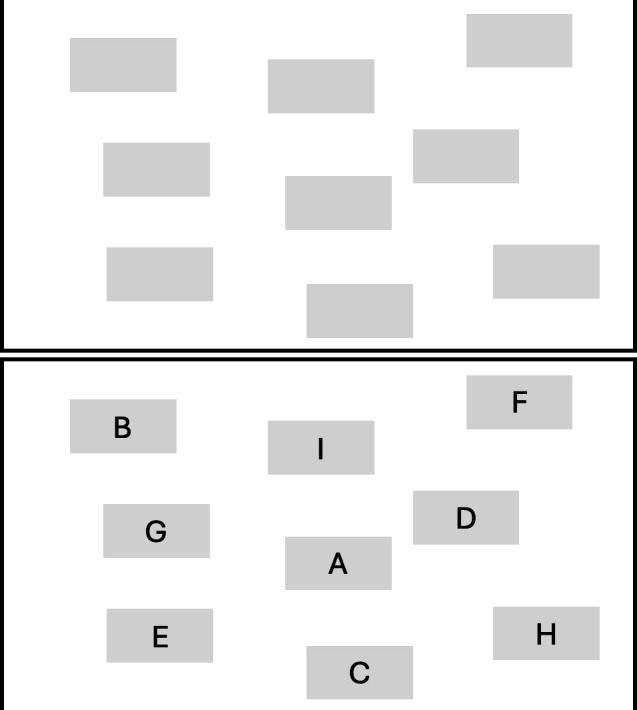
VSSP secondary task. The top image shows the block presentation at the start of each trial. The bottom image shows the blocks at the end of the trial, with letters added to allow the participant to recall the sequence verbally.

A visuospatial span task was used as a secondary task to load the VSSP, which is an adapted version of a Corsi blocks task [73]. Participants were shown nine blue squares on the computer screen (figure 1). The blocks then changed colour individually (changing red for 1 second, then reverting to blue), which indicated a sequence (see video on OSF: https://bit.ly/3lFeWll). The blocks remained in the same positions on the screen for the length of the experiment. As in the verbal secondary task, sequence length ranged from three to seven items and increased throughout each condition of the primary task, with four trials for each span length.

After completing the primary task, the blocks were presented again. It was important that participants responded in the same manner in both the PL and VSSP dual-task conditions, so that they are comparable. It was also important that the response modes for the primary and secondary tasks were different, to ensure that we were isolating the processing mode rather than the response mode. Participants responded to the primary task with their hands and, therefore, participants responded verbally to the secondary tasks.

To allow participants to respond verbally, each square was labelled with a letter and the participant indicated to the experimenter the order of the sequence in reverse (figure 1). The location of the letters was randomly generated for each trial. This prevented participants from using their PL to rehearse the visual sequence while completing the primary task because the phonological response mechanism was only involved during recall. Again, for the secondary task, we recorded the accuracy of recall.

### 2.4. Procedure

**Figure 2.**
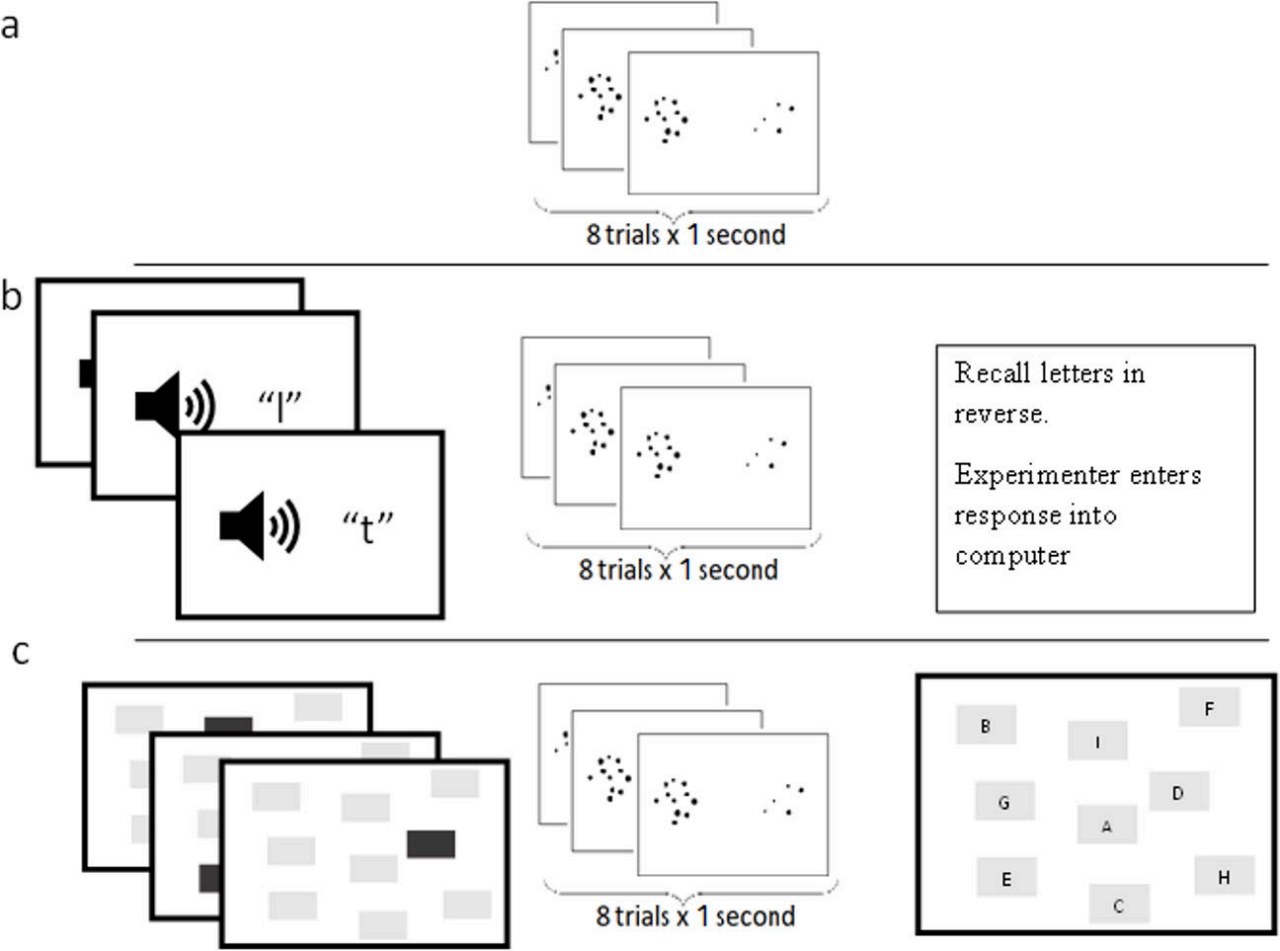
Example of conditions for non-symbolic comparison. Condition (a) shows standalone primary, (b) shows phonological dual-task and (c) shows visuospatial dual-task.

All participants completed all conditions across two sessions. The order of primary tasks was counterbalanced across participants. The order of secondary tasks was also counterbalanced across participants but remained constant for individual participants across the two sessions.^1^ This means that participants completed each primary task in standalone and dual-task conditions, before moving on to the next primary task. Participants also completed both secondary tasks as standalone. An example of the procedure demonstrated for the non-symbolic comparison condition is shown in figure 2.

### 2.5. Data analysis

A factor to note when analysing dual-task performance is the trade-off between primary and secondary performance. Therefore, for each research question, we examined performance in both tasks, in comparison to the standalone conditions.

First, for each participant, we calculated the mean accuracy and the median RT (for correct trials only) for the primary task and the accuracy in the secondary task. Before conducting our main analysis, we performed normality checks. We plotted the data and examined skewness and kurtosis values. Following recommendations (e.g. [74]), we conducted non-parametric paired comparisons (Wilcoxon signed-rank) instead of parametric paired *t*-tests if skew was >|3| or kurtosis was >|4|. Outliers were examined for performance on each task (i.e. primary and secondary tasks) and extreme outliers (>3.29 s.d. [75]) were removed from the respective analyses. All analyses were conducted using R Statistical Software (v. 4.2.2 [76]).

Research questions 1 and 2 were answered via a series of planned paired comparisons to look for (a) differences in primary task performance between standalone and dual-task conditions and (b) differences in secondary task performance between standalone and dual-task (interference) conditions. These were performed separately for the different primary tasks (non-symbolic comparison, symbolic comparison, cross-modal comparison) and secondary tasks (PL, VSSP). Analyses were conducted for all trials combined before further analyses considered small and large trials separately, to answer research question 3. A summary of the pre-registered analysis plan can be found in appendix A, the detailed analysis in appendix C and the study’s Stage 1 registered report on OSF (https://osf.io/z9cv2).

## 3. Results

After the removal of extreme outliers for each individual variable, we inspected the descriptive statistics for accuracy and median RT on all the primary tasks (symbolic, non-symbolic and cross-modal) for all trials combined (table 1) and small and large quantities separately (table 2) for the standalone and each interference (PL or VSSP) condition.

**Table 1.** Descriptive statistics for primary and secondary task performance (accuracy and RT in seconds) in all experimental conditions. Note: PL = phonological, VSSP = visuospatial.

		*N*	mean (s.d.)	min.	max.	skewness	kurtosis
non-symbolic primary task standalone	accuracy	80	0.94 (0.04)	0.78	1	−1.48	2.77
	median RT	79	0.53 (0.09)	0.37	0.81	0.64	0.18
non-symbolic primary task with PL secondary task	accuracy	80	0.94 (0.04)	0.83	0.99	−1.24	2.99
	median RT	79	0.55 (0.09)	0.38	0.82	0.80	0.31
non-symbolic primary task with VSSP secondary task	accuracy	80	0.93 (0.04)	0.77	0.99	−1.45	2.98
	median RT	79	0.54 (0.09)	0.38	0.82	1.03	1.46
symbolic primary task standalone	accuracy	79	0.97 (0.03)	0.87	1	−1.29	2.31
	median RT	79	0.47 (0.08)	0.37	0.75	0.91	0.86
symbolic primary task with PL secondary task	accuracy	81	0.97 (0.02)	0.89	1	−1.39	1.82
	median RT	79	0.50 (0.07)	0.37	0.69	0.49	−0.11
symbolic primary task with VSSP secondary task	accuracy	81	0.97 (0.03)	0.86	1	−2.14	5.92
	median RT	79	0.50 (0.07)	0.38	0.68	0.49	−0.07
cross-modal primary task standalone	accuracy	80	0.91 (0.05)	0.76	1	−0.85	0.61
	median RT	79	0.63 (0.12)	0.4	1.16	1.81	5.85
cross-modal primary task with PL secondary task	accuracy	79	0.90 (0.07)	0.66	1	−1.32	3.11
	median RT	79	0.63 (0.12)	0.42	1.17	1.74	5.11
cross-modal primary task with VSSP secondary task	accuracy	79	0.90 (0.05)	0.70	1	−0.87	1.40
	median RT	79	0.63 (0.10)	0.4	0.92	0.76	0.73
secondary PL task accuracy	standalone	81	0.51 (0.22)	0	0.95	0.02	−0.51
	with non-symbolic primary task	81	0.51 (0.21)	0	1	−0.05	−0.17
	with symbolic primary task	81	0.50 (0.24)	0.05	1	0.28	−0.89
	with cross-modal primary task	81	0.51 (0.22)	0	0.9	−0.23	−0.69
secondary VSSP task accuracy	standalone	81	0.59 (0.60)	0	1	−0.61	0.99
	with non-symbolic primary task	81	0.40 (0.20)	0	0.95	0.18	−0.36
	with symbolic primary task	81	0.47 (0.21)	0	1	0.21	−0.4
	with cross-modal primary task	81	0.38 (0.23)	0	0.85	0.22	−0.8

**Table 2.** Descriptive statistics for primary and secondary task performance (accuracy and RT in seconds) in all experimental conditions for small and large quantities separately. Note: PL = phonological, VSSP = visuospatial.

		small quantities						large quantities					
		*N*	mean (s.d.)	min.	max.	skewness	kurtosis	*N*	mean (s.d.)	min.	max.	skewness	kurtosis
non-symbolic primary task standalone	accuracy	80	0.96 (0.04)	0.79	1	−2.49	4.45	80	0.88 (0.07)	0.70	1	−0.57	−0.23
	median RT	79	0.51 (0.08)	0.38	0.73	0.57	−0.15	79	0.59 (0.13)	0.35	1	0.82	0.87
non-symbolic primary task with PL secondary task	accuracy	80	0.97 (0.03)	0.85	1	−1.76	3.34	80	0.89 (0.07)	0.72	1	−0.66	−0.12
	median RT	79	0.53 (0.09)	0.37	0.79	0.77	0.18	79	0.60 (0.13)	0.34	0.97	0.94	0.80
non-symbolic primary task with VSSP secondary task	accuracy	80	0.96 (0.04)	0.81	1	−1.77	4.45	80	0.88 (0.07)	0.63	1	−1.07	1.61
	median RT	79	0.53 (0.08)	0.38	0.80	1.04	1.84	79	0.59 (0.11)	0.37	0.88	0.83	0.56
symbolic primary task standalone	accuracy	79	0.98 (0.02)	0.91	1	−1.22	0.99	79	0.94 (0.05)	0.75	1	−1.03	1.47
	median RT	79	0.47 (0.07)	0.37	0.71	0.79	0.35	79	0.50 (0.09)	0.37	0.80	0.88	0.63
symbolic primary task with PL secondary task	accuracy	80	0.98 (0.02)	0.94	1	−0.91	1.23	81	0.95 (0.05)	0.80	1	−1.42	2.13
	median RT	79	0.49 (0.07)	0.36	0.66	0.48	−0.10	79	0.52 (0.09)	0.38	0.77	0.54	0.09
symbolic primary task with VSSP secondary task	accuracy	81	0.99 (0.02)	0.89	1	−2.17	7.52	80	0.95 (0.05)	0.80	1	−1.15	1.04
	median RT	79	0.48 (0.07)	0.37	0.67	0.57	0.11	79	0.53 (0.08)	0.39	0.73	0.43	−0.34
cross-modal primary task standalone	accuracy	80	0.92 (0.05)	0.78	1	−0.61	6.00	80	0.89 (0.08)	0.63	1	−0.83	0.75
	median RT	79	0.61 (0.12)	0.39	1.12	1.96	6.75	79	0.68 (0.14)	0.41	1.20	1.43	3.04
cross-modal primary task with PL secondary task	accuracy	78	0.92 (0.05)	0.75	1	−0.99	1.11	81	0.86 (0.09)	0.63	1	−0.89	0.39
	median RT	79	0.62 (0.11)	0.42	1.06	1.37	2.96	79	0.66 (0.14)	0.42	1.26	1.98	5.82
cross-modal primary task with VSSP secondary task	accuracy	79	0.92 (0.05)	0.75	1	−1.20	1.95	80	0.87 (0.10)	0.60	1	−1.09	1.09
	median RT	79	0.62 (0.10)	0.4	0.94	0.82	1.09	79	0.67 (0.11)	0.43	1.00	0.80	0.87

Subsequently, we ran the analyses addressing our three research questions as outlined in our detailed analysis plan (appendix C). Note that for some bivariate comparisons, the data did not exceed kurtosis limits once all relevant exclusions had been performed and therefore a *t*‐test was performed even if the individual variables exceeded this limit.

### 3.1. RQ1: are Arabic symbols and non-symbolic representations accessed automatically or does access require the involvement of working memory components?

#### 3.1.1. RQ1a: symbolic comparison primary task

**Figure 3.**
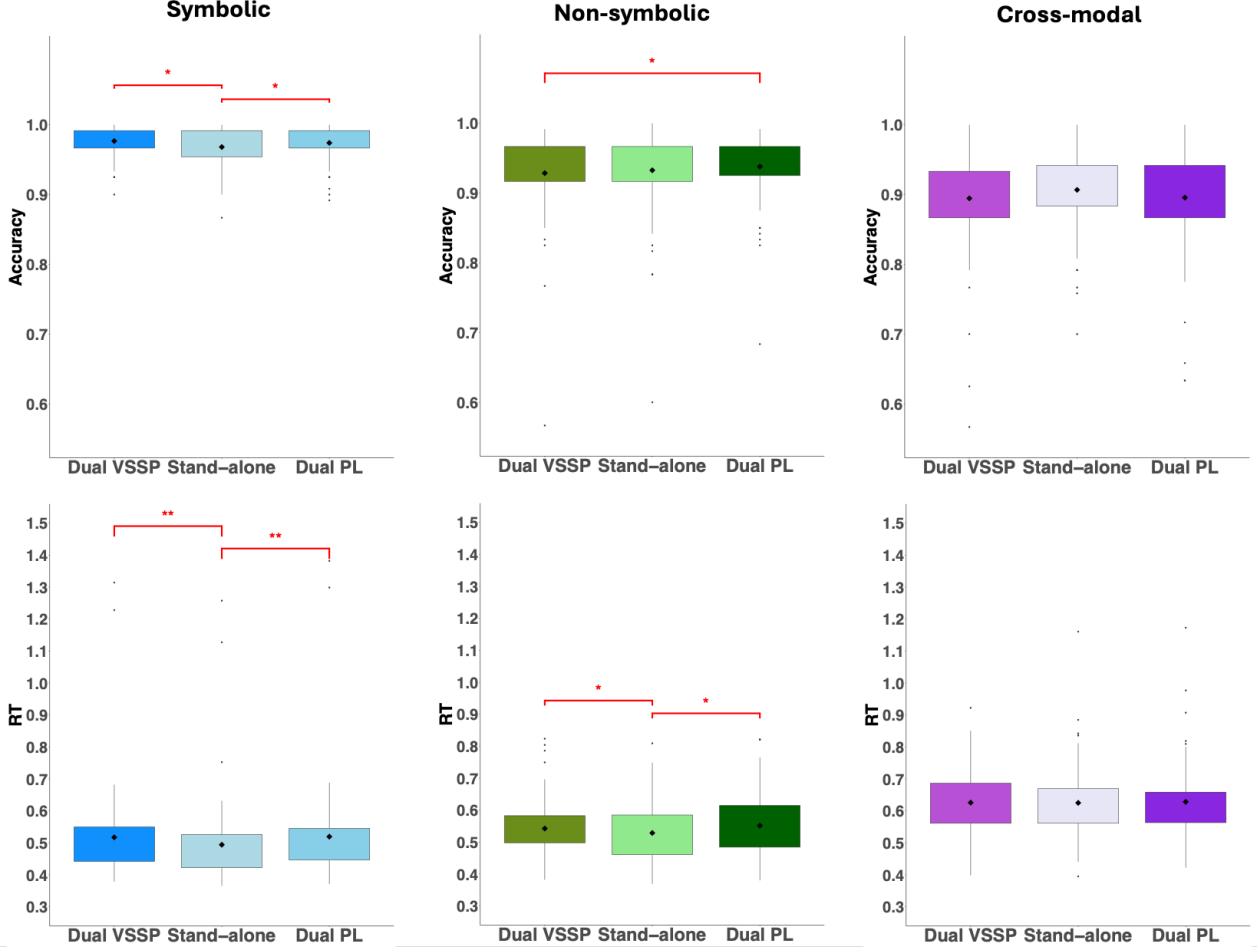
Accuracy and median RT (seconds) on the three primary tasks (symbolic, non-symbolic and cross-modal) for standalone and interference (dual-task) conditions. Paired comparison results are indicated by *p < 0.05, **p < 0.001.

We ran six paired sample *t*-tests focusing on the symbolic comparison primary task (figure 3). The *t*‐test comparing participants’ accuracy in symbolic comparison with and without PL interference demonstrated a significant difference, *t*(78) = 1.98, *p* = 0.05, *d* = 0.22, however, in the opposite direction to our expectations. Specifically, participants performed better with PL interference than without. We also found a significant difference, *t*(78) = 4.55, *p* < 0.001, *d* = 0.51, when comparing the corresponding median RT data although this time in the expected direction, namely participants were slower in the symbolic primary task with PL interference than the one without. Contrary to our predictions, the paired sample *t*-tests comparing symbolic performance with and without VSSP interference demonstrated significant differences with both the accuracy, *t*(78) = 2.75, *p* < 0.05, *d* = 0.31, and median RT data, *t*(78) = 4.23, *p* < 0.001, *d* = 0.48, with better accuracy but slower performance in the VSSP dual-task compared to the standalone condition. Finally, comparing the two interference conditions (i.e. symbolic primary task with PL dual-task versus symbolic primary task with VSSP dual-task), we found no difference for either accuracy, *t*(80) = 0.58, *p* = 0.57, *d* = 0.06, or median RT, *t*(78) = 0.09, *p* = 0.93, *d* = 0.01.

**Figure 4.**
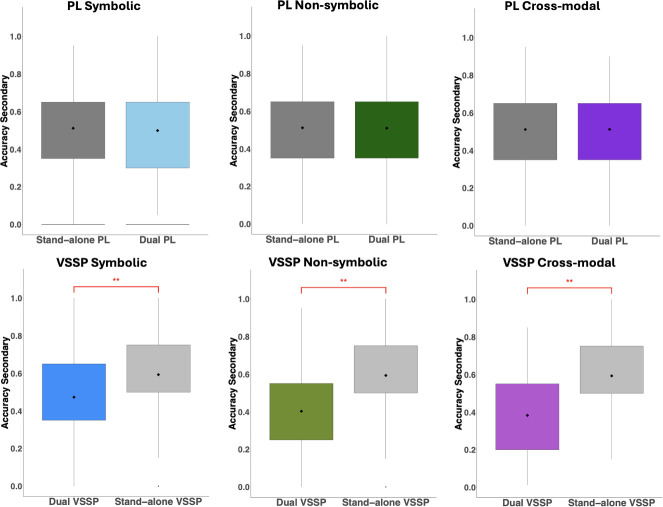
Accuracy on the PL and VSSP secondary tasks conducted standalone versus dual-task conditions (symbolic, non-symbolic and cross-modal). Paired comparison results are indicated by *p < 0.05, **p < 0.001*.*

Turning now to secondary task performance (figure 4), with the symbolic primary task, we found no difference between participants’ accuracy on the PL secondary task between the standalone and the interference (dual-task) condition, *t*(80) = 0.59, *p* = 0.56, *d* = 0.07*,* but there was a significant difference between participants’ accuracy on the VSSP secondary task conducted standalone versus the symbolic dual-task condition, *t*(80) = 5.31, *p* < 0.001, *d* = 0.59, with participants performing better in the standalone VSSP compared with the symbolic dual-task condition.

#### 3.1.2. RQ1b: non-symbolic comparison primary task

We ran six paired sample *t*-tests focusing on the non-symbolic comparison primary task (figure 3). As expected, the comparison between the non-symbolic task conducted with and without PL interference showed no significant difference in accuracy, *t*(78) = 1.00, *p* = 0.32, *d* = 0.11; however, surprisingly, there was a significant difference in the median RT data, *t*(78) = 3.27, *p* = 0.002, *d* = 0.37, with participants being slower in the interference compared with the standalone condition. The paired sample *t*-tests comparing non-symbolic with and without VSSP interference demonstrated no difference with the accuracy data, *t*(79) = 0.90, *p* = 0.37, *d* = 0.10, but a significant difference with the median RT data, *t*(78) = 2.22, *p* = 0.03, *d* = 0.25, with participants being slower in the VSSP interference compared with the standalone condition. Finally, performance in the two non-symbolic interference conditions (PL and VSSP) significantly differed, *t*(79) = 2.44, *p* = 0.02, *d* = 0.27, with participants performing better under PL than VSSP interference. Median RTs did not differ between these two conditions, *t*(78) = 1.48, *p* = 0.14, *d* = 0.17.

Turning our focus now to the secondary tasks (figure 4), as expected, we found no difference between the PL secondary performed standalone versus the non-symbolic interference condition, *t*(80) = 0.11, *p* = 0.91, *d* = 0.01; however, once again participants’ accuracy significantly dropped in the VSSP secondary task in the non-symbolic interference versus the standalone condition, *t*(80) = 8.80, *p* < 0.001, *d* = 0.98.

### 3.2. RQ2: can adults translate between Arabic and non-symbolic representations automatically or does this require access to verbal representations?

Once again, we ran six paired sample *t*-tests this time focusing on the cross-modal comparison primary task (figure 3). The comparison between the cross-modal with and without PL interference revealed no significant difference in accuracy, *t*(77) = 1.64, *p* = 0.10, *d* = 0.19, or median RTs, *V* = 1566, *p* = 0.90, *d* = 0.04 (non-parametric Wilcoxon signed-rank). There was also no difference between the cross-modal with and without VSSP interference in accuracy, *t*(77) = 1.94, *p* = 0.056, *d* = 0.22, or median RT, *V* = 1597, *p* = 0.78, *d* = 0.01 (non-parametric Wilcoxon signed-rank). Furthermore, we found no difference between the two interference conditions (PL and VSSP) in accuracy, *t*(77) = 0.67, *p* = 0.50, *d* = 0.08, or median RT, *V* = 1497, *p* = 0.69, *d* = 0.04 (non-parametric Wilcoxon signed-rank).

The expected involvement of WM in cross-modal comparison was found when examining participants’ secondary task performance (figure 4). Specifically, we found that participants were more accurate in the VSSP secondary task when performed standalone compared to its cross-modal dual-task counterpart, *t*(80) = 10.34, *p* < 0.001, *d* = 1.15. However, there was no significant difference between the standalone PL secondary and its cross-modal dual-task counterpart *t*(80) = 0.03, *p* = 0.97, *d* = 0.003.

### 3.3. RQ3: does the processing of numerical information differ for small and large quantities?

#### 3.3.1. RQ3a: symbolic and cross-modal comparison

For symbolic and cross-modal comparison, we hypothesized PL involvement and no difference in the effect of PL interference for small and large numbers. In symbolic comparison, paired sample *t*-tests comparing small-number trials with and without PL interference demonstrated a significant difference in median RT, *t*(78) = 4.70, *p* < 0.001, *d* = 0.53, but not in accuracy, *t*(77) = 0.69, *p* = 0.49, *d* = 0.08. As expected, participants were slower in the small-number trials in the PL interference compared to standalone condition. For large quantities, the same comparison revealed a significant difference between with and without PL interference in both accuracy, *t*(78) = 2.35, *p* = 0.02, *d* = 0.26, and median RT, *t*(78) = 3.49, *p* < 0.001, *d* = 0.39. For large-number trials, participants performed better but slower in the interference condition compared to their standalone counterparts.

For cross-modal comparison, we found no difference for the small-quantity trials with and without PL interference (accuracy: *t*(76) = 0.42, *p* = 0.68, *d* = 0.05; median RT: *V* = 1332, *p =* 0.30, *d* = 0.13—non-parametric Wilcoxon signed-rank). However, we found a significant difference in the corresponding large-quantity trials with both accuracy (*t*(79) = 2.72, *p* = 0.008, *d* = 0.30) and median RT (*V* = 1952, *p* = 0.04, *d* = 0.16—non-parametric Wilcoxon signed-rank). Participants performed better in the cross-modal large-quantity trials without PL interference but faster in the corresponding trials with PL interference.

#### 3.3.2. RQ3b: non-symbolic comparison

**Figure 5.**
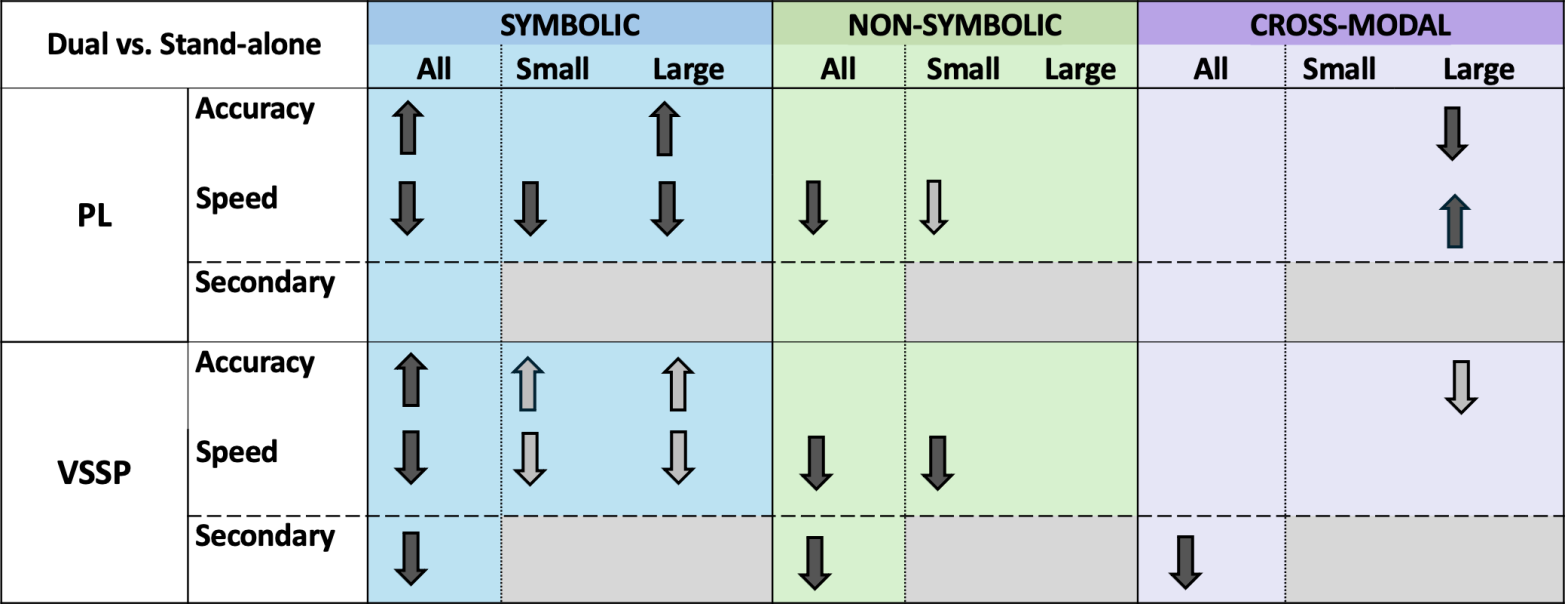
Overview of all significant comparisons (*p* < 0.05) across the experimental conditions of the three number comparison conditions (symbolic, non-symbolic and cross-modal). Black arrows depict the pre-registered comparisons, and grey arrows the exploratory comparisons (see appendix D). Arrows facing upwards indicate better accuracy or faster speed in the interference condition (dual-task) whereas arrows facing downwards indicate reduced accuracy or slower speed in the interference condition (dual-task).

For the case of non-symbolic comparison, we hypothesized a difference in the effects of VSSP interference between small and large quantities. For small quantities, paired sample *t*-tests comparing trials with and without VSSP interference demonstrated no difference in accuracy (*V* = 1193.5, *p* = 0.20, *d* = 0.08; non-parametric Wilcoxon signed-rank), but a significant difference in median RT, namely participants were slower in the dual-task small quantity trials compared to the standalone small quantity trials (*t*(78) = 2.70, *p* = 0.009, *d* = 0.30). However, we found no difference between the large quantities with and without VSSP interference (accuracy: *t*(79) = 0.71, *p* = 0.47, *d* = 0.08; median RT: *t*(78) = 0.17, *p* = 0.87, *d* = 0.02). Figure 5 summarizes all the planned comparisons (black arrows) addressing our research questions as outlined above.

## 4. Discussion

Basic number processing is defined as the ability to understand, estimate or discriminate between numerical magnitudes [5,77]. The present study examined whether adults process numbers presented in different modalities and translate between them automatically or if doing so requires WM resources. Participants conducted non-symbolic, symbolic and cross-modal comparison tasks with and without PL and VSSP WM interference. Interference was implemented by asking participants to perform the numerical comparison tasks while also undertaking a WM task where they had to retain either letters that they heard (PL) or visuospatial patterns that they saw (VSSP) and then recall them backwards. As these secondary tasks required the manipulation of the stored elements, they also involved the CE component of WM, i.e. the limited-capacity attentional system overseeing and coordinating the activities of the PL and VSSP [78–80]. The PL and VSSP WM interference tasks were also completed as standalone tasks. Results demonstrated for the first time that all three types of comparison necessitate WM resources. Symbolic comparison necessitated VSSP WM, i.e. the VSSP component of WM in combination with the CE. Surprisingly, in this case, participants’ accuracy improved under both interference conditions, revealing the positive effects that embedding executive function challenges can have on simple and familiar numerical tasks. Non-symbolic comparison necessitated VSSP WM but also PL albeit to a lesser extent. Under VSSP interference, performance was reduced in both the primary and the secondary task, whereas under PL interference only in the primary and significantly less than the VSSP. Finally, cross-modal comparison primarily taxed VSSP WM processes. Also, we observed different interference effects in processing and translating numerical representations for smaller (1–4) and larger (5–9) numerosities, which were examined separately (so differences in the effects cannot be claimed). Overall, our findings provide clear evidence that basic number processing in adults is not automatic; it necessitates WM resources. Adults require WM resources for both simply processing numerical representations (non-symbolic and symbolic) as well as for translating between them. Our results also demonstrate the flexible and dynamic role that WM plays in basic number processing, aiding in the adaptive utilization of phonological and visuospatial strategies when processing numerical representations. We discuss our findings in more detail and the respective theoretical and methodological implications as follows.

### 4.1. Symbolic magnitude comparison

We start learning and understanding symbols from a very young age and continue to develop our symbolic number skills throughout our lives. It is unsurprising, therefore, that it is intuitively assumed that by adulthood we process symbolic numbers automatically with little conscious effort. The typical symbolic comparison task used to assess basic number processing, where participants see two digits on the screen and must decide which is larger as quickly and as accurately as possible, can be considered effortless for adults. Our dual-task paradigm revealed that WM is actively involved in the flexible processing of numerical representations when undertaking such a symbolic comparison task.

Surprisingly, participants’ accuracy improved instead of dropping under both types of the implemented active CE interference, i.e. with both the PL and VSSP WM interference. This was triggered by the interference conditions. At the same time, participants were slower under both interference conditions compared to the one without. Perhaps with WM interference, which made the task more challenging and potentially less tedious, participants’ focus on the primary task improved, leading to improved accuracy and slower responses. Conversely, the challenge induced by the interference conditions may have slowed participants down, leading to improved performance. Our exploratory analyses (appendix D, table 5) showed a significant but small speed–accuracy trade-off (*r* = 0.20) for the condition with PL interference, but not for the condition with VSSP interference nor the condition without interference. However, these speed–accuracy correlation coefficients did not differ significantly, which may suggest no change in participants’ speed–accuracy trade-off across the conditions. Thus, a speed–accuracy trade-off on its own does not appear to explain the finding of improved accuracy in interference conditions. We should highlight here though that this is only a provisional assumption based on exploratory analyses for which we had not conducted a power analysis, and should be therefore considered tentative. Nevertheless, it is clear that accuracy improved due to the interference conditions, therefore there must be WM involvement. The fact that we found no difference between the two interference conditions (in both accuracy and RT) indicates that both had a similar effect on symbolic comparison. As expected, results were similar when splitting the trials based on the size of the number, i.e. there was no difference in the effect of interference for trials on symbolic comparison with small or large quantities.

The role of WM in symbolic comparison became clearer when examining the trade-off between the primary and the secondary tasks. Participants’ performance dropped in the VSSP secondary task, but not the PL secondary task. Similarly, van Dijk *et al.* [66] and Herrera *et al.* [64], who implemented verbal and visuospatial load on adults’ symbolic magnitude comparison processing, found performance was affected only under the VSSP, not the PL load. In those studies, the WM load only required the storage of elements of information during the dual conditions, whereas the secondary tasks used in the present study also necessitated processing the given visuospatial elements. Thus, our study extends past findings by demonstrating that symbolic comparison necessitates not just VSSP storage but also VSSP processing, i.e. the combination of the VSSP component of WM with the CE. This suggests that participants use VSSP strategies to solve this type of numerical comparison task, for example by using non-symbolic or mental number line representations for the symbolic quantities. These results are in line with the triple-code model [7], i.e. the involvement of VSSP WM may reflect the involvement of the analogue code in symbol–symbol comparison, whereas the lack of evidence for PL WM involvement suggests that the verbal code is not necessary for this type of numerical comparison. Notably, the triple-code model [7] does not mention the potential role that WM may play in mentally representing and processing numerical representations. In Dehaene’s [7] review, WM is only mentioned concerning its role in arithmetic. The present study extends this model by evidencing the key role that WM plays in processing and translating between the different types of numerical representations.

Furthermore, our findings for symbolic comparison suggest that the fact that a task requires little cognitive effort does not mean that it is processed automatically. On the contrary, we observed widespread interference effects in this primary task (figure 5). Embedding executive function challenges in easy tasks can potentially enhance performance. Indeed, in cognitive psychology, there have been other instances where under certain conditions participants’ performance improved under WM load conditions [81]. For example, Makovski *et al*. [82] demonstrated that orienting attention in visual WM under dual-task conditions can lead to improved performance by reducing interference from memory probes. Thus, it appears that attentional mechanisms can mitigate WM interference under certain conditions. Taken together, these findings reveal the complexity of cognitive processes and the potential for adaptive mechanisms to enhance performance even in the presence of interference [81].

### 4.2. Non-symbolic magnitude comparison

In the non-symbolic magnitude comparison task, performance was affected under both WM load interference conditions, with participants being slower under both conditions. Importantly, this time there was a significant difference in the impact of the two interference conditions; performance with the PL interference task was higher than its VSSP counterpart. Finally, participants’ performance again dropped in the VSSP secondary task, but not in the PL secondary task. Taken together, these findings provide clear evidence that both subcomponents of WM, the VSSP and the PL are needed in combination with the CE when processing non-symbolic quantities. In 2014, Xenidou-Dervou *et al.* [27] conducted a dual-task study with young children where they implemented phonological, visual, spatial and CE interference on a non-symbolic approximate arithmetic task, that is a task where participants see two dot arrays dropping inside a box on one side of the screen before having to make the judgment on whether the sum of the arrays was larger or smaller than a third dot array. They found that performance dropped only under the CE interference condition, where participants had to process phonological information while undertaking the primary task. We find this PL–CE combination to be crucial for simple non-symbolic magnitude comparison in adults too. Notably, in Xenidou-Dervou *et al*. [27], the non-symbolic task entailed only large quantities (6 up to 70 dots) and the visual and spatial secondary tasks only necessitated storage of the corresponding elements. Thus, the involvement of the VSSP–CE combination, i.e. VSSP WM, was not directly examined, although it can be argued that to an extent dual tasks inherently necessitate executive functions (such as switching between two tasks) [83]. The findings of the present study clearly demonstrate that visuospatial processing is not only necessary but also more important than phonological processing for non-symbolic magnitude comparison.

Another interesting finding arose when considering the size of the quantities involved in the trials. Contrary to our hypotheses, when considering only primary task performance, the VSSP interference was observed only in the small-quantity trials, not the large (although the involvement of the VSSP overall is still evident from the secondary-task results). Given the unexpected involvement of the PL in non-symbolic comparison, we opted to conduct similar exploratory analyses splitting the trials based on the size of the quantities under the PL interference condition too and found the PL to be involved again only in the small-quantity trials. This pattern of results in the small-quantity trials is the same as that observed for the symbolic comparison task, tentatively supporting the assumption that small non-symbolic quantities may be processed similarly to symbolic representations [36].

### 4.3. Cross-modal magnitude comparison

For the cross-modal primary task, we hypothesized that the translation between symbolic and non-symbolic representations would require access to verbal representations and therefore the PL. Surprisingly, however, participants’ performance on the cross-modal comparison primary task was not affected by either WM interference conditions. Instead, their performance dropped once again in the VSSP secondary task, but not the PL secondary task, revealing yet again the crucial role that visuospatial processing plays not only in processing numerical representations but also in translating between them. The finding that VSSP, but not PL, WM is necessary for cross-modal comparison addresses an open question in the triple-code model [7]; it suggests that translating from non-symbolic to symbolic representations is possible without accessing the verbal code.

Interestingly, we found some evidence of potential involvement of the PL only for the large-quantity trials, which suggests that translation between symbolic and non-symbolic representations may require the processing of verbal representations only for large quantities. However, these results are not conclusive. Although accuracy dropped in the cross-modal large-quantity trials, unexpectedly corresponding RT dropped too (i.e. participants were faster under PL WM load) and the difference between the two interference conditions was not significant, indicating overall additional WM load from completing two tasks in these trials, i.e. CE involvement.

### 4.4. Overview

The present study’s findings extend our understanding of the causal mechanisms that drive basic number processing skills and the fundamental role that WM processing plays herein. We found that all three types of comparison employ WM resources: symbolic and cross-modal involve VSSP WM, whereas non-symbolic comparison involves both VSSP and PL WM. We had hypothesized that numerical processing would necessitate not just the mere storage of numerical representations in WM but also their processing; therefore, we used secondary tasks that would tax the CE component as well as the storage components of WM. Indeed, we found that although the different forms of numerical processing may differ on the type of subcomponent that they may employ (PL or VSSP), therefore reflecting the type of strategies an individual may use to undertake the task at hand, i.e. phonological or visuospatial, we see that all forms of numerical processing necessitated the CE one way or another, i.e. the processing of the different types of numerical representations.

Interestingly, and unexpectedly, we found that our interference conditions affected the harder task of translating between modalities, i.e. cross-modal comparison, in fewer performance outcomes compared to the easier symbolic and non-symbolic comparison tasks. It is well known that translating between numerical representations is harder than non-symbolic processing and this in turn is harder than symbolic processing [44]. We confirmed this to be the case in our data too (see exploratory analyses in appendix D). Inspecting figure 5, which provides an overview of all the performance outcomes that were affected (or not) by the WM load conditions, one notices the most effects in symbolic comparison and the least in cross-modal comparison. This is the opposite of what one may have intuitively expected as we tend to think in terms of the extent of reduced performance due to WM load in a particular component, not across the breadth of available cognitive resources. What this bird’s eye view of the overall results demonstrates is that when engaging in more difficult tasks such as translating between numerical representations, individuals may use their WM resources less flexibly. This phenomenon is typically supported by research showing that extensive mental effort can lead to the depletion of our limited WM resources, resulting in decreased performance compared to tasks requiring less mental effort (e.g. [84]). Our findings extend this even to cross-modal number comparison, which although may be more taxing compared to the other notation-specific comparison tasks used (symbolic or non-symbolic), it is not that arduous in relative terms. It is in fact considered a very basic number-processing skill for adults. Accumulating these findings and assumptions, perhaps we should not view WM only in terms of a bottleneck—a limited-capacity mental space perspective that considers harder tasks taking up more cognitive resources, but rather that harder tasks lead to less flexible/adaptable usage of an individual’s available WM resources.

### 4.5. Limitations and future directions

The dual-task paradigm is a robust experimental design, which allows for causal conclusions to be drawn regarding underlying mechanisms [63]. The present study has many methodological strengths; we used active WM interference conditions which allowed us to examine the trade-off between primary and secondary tasks, differential response modalities for primary and secondary tasks, powered our study adequately, pre-registered methods and analytical approach. However, our approach is not free of limitations. First, our design is limited when it comes to understanding the functions of the CE, which we found to be a crucial component [83]. Future studies should strive to unravel the specific functions of the CE involved in number processing and the conditions under which attentional mechanisms and executive function challenges can mitigate WM interferences and even enhance performance. Second, although the secondary tasks that we used can be seen as a strength as we extend previous studies, which had only used simple WM load conditions that did not require processing of information in one’s WM, future studies should directly contrast the effects of storage versus processing. Given the prominent role of the VSSP component in all forms of basic number processing in our study, future research should zoom into this component and examine the role of the visual and spatial subcomponents separately [27,85]. Finally, our study allows limited conclusions regarding the comparison of the effects of WM load on small or large numbers/quantities. That is because WM load was imposed before and after the presentation of eight trials of the primary task, where both small and large numbers could have been shown. Future research should use a blocked design where the WM interference is imposed separately for small and large trials for a more robust comparison.

Finally, beyond the theoretical and methodological implications, this study’s findings also generate an interesting practical implication: even a simple task such as comparing small numbers or quantities may be more taxing to one’s limited WM resources than intuitively expected. From an educational perspective, future research should examine how taxing numerical processing is for children, who are still in the process of learning and developing their numerical skills, and the conditions under which increasing or decreasing the WM demands of a numerical task may benefit or hinder performance and learning.

### 4.6. Concluding remarks

How we process numerical representations is a fundamental question in the field of numerical cognition. The present study’s findings extend our theoretical understanding by revealing that although numerical representations may be activated automatically (e.g. [47,48,86]), when processing is needed WM resources are employed. WM resources are required both for simply processing numerical representations (non-symbolic and symbolic comparison) as well as for translating between them (cross-modal comparison). The flexible use of visuospatial strategies seems to be most crucial for adults’ basic number processing, confirming the strong link between numbers and space in numerical cognition [87].

## Data Availability

Raw data, materials and analysis scripts are available on the Open Science Framework.

## Appendix A

See table 3.

**Table 3.** Calculations of effect sizes.

primary condition	analysis	standalone mean (s.d.)	minimal effect of interest (number of trials difference)	minimal effect size of interest (Cohen’s *d*)	resulting number of participants
symbolic comparison	primary accuracy	99.6% (1.88)	5 trials	1.66	5
non-symbolic comparison	primary accuracy	99.7% (0.3)	5 trials	0.35	72
cross-modal comparison	primary accuracy	87.6% (9.38)	5 trials	0.33	81
primary condition	analysis	standalone mean (s.d.)	minimal effect detectable with 81 participants (number of trials difference or increase in RT)		
symbolic comparison	primary RT	401 ms (127)	50 ms		
	secondary (PL) accuracy	16 (4)	1.3 sequences		
non-symbolic comparison	primary RT	499 ms (141)	50 ms		
	secondary (VSSP) accuracy	12 (4)	1.3 sequences		
cross-modal comparison	primary RT	799 ms (244)	80 ms		
	secondary (PL) accuracy	16 (4)	1.3 sequences		

## Appendix C

### Detailed analysis plan

#### Preliminary analyses

For each participant, mean accuracy will be calculated for the primary task and secondary task. Median RT (for correct trials only) will be calculated for the primary task conditions. Prior to conducting our main analysis (table 4), we will perform normality checks. Data will be plotted, and skewness and kurtosis values will be examined. We expect some level of skew for the accuracy data (particularly for the Arabic digit condition) due to high accuracies in the standalone condition. Following recommendations (e.g. [47]), we will conduct non-parametric paired comparisons (Wilcoxon signed-rank) instead of parametric paired *t*-tests if skew is >|3|or kurtosis is >|4|. Outliers will be examined for performance on each task (i.e. primary and secondary tasks). Extreme outliers (>3.29 s.d. [25]) will be removed from the analysis. All analyses were conducted in R.

**Table 4.** Detailed analysis plan.

question	hypothesis	sampling plan	analysis plan	rationale for deciding the sensitivity of the test for confirming or disconfirming the hypothesis	interpretation given different outcomes	theory that could be shown wrong by the outcomes
**RQ1a.** Is the processing of Arabic digits automatic, or does it require the involvement of WM components?	**RQ1a**. Processing of Arabic digits will require the involvement of the PL but not the VSSP. This means we will expect to see a difference in performance between the **symbolic primary task** in the PL dual-task condition compared to the VSSP dual-task condition and standalone condition, OR a difference between the **PL secondary task** in the dual-task condition when compared to the PL standalone condition	based on the effect size calculations described above, the smallest effect size of interest for this RQ is 0.5. Using a power level of 90%, an alpha level of 0.05 and a minimum effect size of Cohen’s *d* = 0.5, the minimum sample required for this RQ is 36	primary tasks **symbolic comparison (accuracy**)paired sample *t*‐test comparing: symbolic primary task with PL dual-task versus symbolic primary task standalone symbolic primary task with PL dual-task versus symbolic primary task with VSSP dual-task symbolic primary task with VSSP dual-task versus symbolic primary task standalone **symbolic comparison (RT**) paired sampled *t*-tests comparing: symbolic primary task with PL dual-task versus symbolic primary task standalone symbolic primary task with PL dual-task versus symbolic primary task with VSSP dual-task symbolic primary task with VSSP dual-task versus symbolic primary task standalone secondary tasks paired samples *t*‐test (standalone PL secondary task versus PL secondary task during dual-task accuracy; standalone VSSP secondary task versus VSSP secondary task during dual-task accuracy)	the calculated effect sizes represent: a reduction in primary accuracy of 5 trials, or a reduction in secondary accuracy of 2 trials. These effect sizes were selected as our smallest effect size of interest based on adults’ performance of the primary and secondary tasks in prior research [44,65]	primary tasks if *t*‐test is significant at *p* < 0.05, and indicates a difference in performance between the PL dual-task condition versus its standalone version and the VSSP dual-task condition, we will look at the means to conclude if the PL is involved in the processing of Arabic digits if *t*‐test is significant at *p* < 0.05, and indicates that performance is different in the PL dual-task condition versus the standalone condition but the *t*‐test indicates that there is no significant difference between the PL dual-task condition and the VSSP dual-task condition then we will conclude that there is an additional WM load from completing two tasks simultaneously; however, we cannot be specific about the component of WM involved secondary tasks if performance in the secondary task is significantly different between the PL dual-task condition and the PL standalone condition, we will look at the means to infer if the PL is involved in the processing of Arabic digits if performance in the secondary task is significantly different between the VSSP dual-task condition and the VSSP standalone condition, we will look at the means to infer if the PL is involved in the processing of Arabic digits	if we observe PL involvement in either the primary or secondary task analysis, we will conclude that participants use verbal labels in the processing of Arabic digits if we observe VSSP involvement in either the primary or secondary task analysis, we will conclude that participants use visual strategies in the processing of Arabic digits if we involve both VSSP and PL involvement, we will conclude that WM is required in processing Arabic digits; however, we cannot be specific about which component if we observe no WM involvement (either PL or VSSP), we will conclude that Arabic digits may be processed automatically
**RQ1b.** Is the processing of non-symbolic representations automatic or does it require the involvement of WM components?	**RQ1a**. Processing of non-symbolic representations will require the involvement of the VSSP but not the PL. This means we will expect to see a difference in performance in the **non-symbolic primary task** in the VSSP dual-task condition compared to the PL dual-task condition and standalone condition, OR a difference between the **VSSP secondary task** in the dual-task condition when compared to the VSSP standalone condition	based on the effect size calculations described above, the smallest effect size of interest for this RQ is 0.35. Using a power level of 90%, an alpha level of 0.05 and a minimum effect size of Cohen’s *d* = 0.35, the minimum sample required for this RQ is 72	primary tasks **non-symbolic comparison (accuracy**) paired sample *t*‐test comparing: non-symbolic primary task with VSSP dual-task versus non-symbolic primary task standalone non-symbolic primary task with VSSP dual-task versus non-symbolic primary task with PL dual-task non-symbolic primary task with PL dual-task versus non-symbolic primary task standalone **non-symbolic comparison (RT**) paired sample *t*‐test comparing: non-symbolic primary task with VSSP dual-task versus non-symbolic primary task standalone non-symbolic primary task with VSSP dual-task versus non-symbolic primary task with PL dual-task non-symbolic primary task with PL dual-task versus non-symbolic primary task standalone secondary tasks paired samples *t*‐test (standalone VSSP secondary task versus VSSP secondary task during dual-task accuracy, standalone PL secondary task versus PL secondary task during dual-task accuracy)		primary tasks if *t*‐test is significant at *p* < 0.05, and indicates that performance is different between the VSSP dual-task condition, the standalone condition and the PL dual-task condition, we will look at the means to determine if the VSSP but not the PL is involved in the processing of non-symbolic quantities if *t*‐test is significant at *p* < 0.05, and indicates that performance is different between the VSSP dual-task condition and the standalone condition but the *t*‐test indicates that there is no significant difference between the VSSP dual-task condition and the PL dual-task condition, then we will conclude that thereis an additional WM load from completing two tasks simultaneously; however, we cannot be specific about the component of WM involved secondary tasks if performance in the secondary task is significantly different in the VSSP dual-task condition than in the VSSP standalone condition, we will look at the means to determine if the VSSP is involved in the processing of non-symbolic quantities if performance in the secondary task is significantly different between the PL dual-task condition and the PL standalone condition, we will determine if the PL is involved in the processing of non-symbolic quantities	if we observe PL involvement in either the primary or secondary task analysis we will conclude that participants use verbal labels in the processing of Arabic digits if we observe VSSP involvement in either the primary or secondary task analysis, we will conclude that participants use visual strategies in the processing of non-symbolic quantities if we observe both VSSP and PL involvement, we will conclude that WM is required in processing non-symbolic quantities; however, we cannot be specific about which component if we observe no WM involvement (either PL or VSSP), we will conclude that non-symbolic quantities may be processed automatically
**RQ2.** Can adults translate between Arabic and non-symbolic representations automatically or does this require access to verbal representations?	translation between Arabic and non-symbolic representations will require the involvement of the PL. This means we will expect to see a difference in performance in the **cross-modal primary task** in the PL dual-task condition compared to the VSSP dual-task condition and standalone condition, OR a difference between the PL secondary task in the dual-task condition when compared to the PL standalone condition	based on the effect size calculations described above, the smallest effect size of interest for this RQ is 0.33. Using a power level of 90%, an alpha level of 0.05 and a minimum effect size of Cohen’s *d* = 0.33, the maximum sample required for this RQ is 81	primary tasks **cross-modal comparison (accuracy**) paired sample *t*‐test comparing: cross-modal comparison with PL dual-task versus cross-modal standalone cross-modal comparison with PL dual-task versus cross-modal with VSSP dual-task cross-modal comparison with VSSP dual-task versus cross- modal comparison standalone **cross-modal comparison (RT**) paired sample *t*‐test comparing: cross-modal comparison with PL dual-task versus cross-modal standalone cross-modal comparison with PL dual-task versus cross-modal with VSSP dual-task cross-modal comparison with VSSP dual-task versus cross-modal comparison standalone secondary tasks paired samples *t*‐test (standalone PL versus dual-task PL accuracy; standalone VSSP versus dual-task VSSP accuracy)	the calculated effect sizes represent either: a reduction in primary accuracy of 5 trials, or a reduction in secondary accuracy of 2 trials	primary tasks if *t*‐test is significant at *p* < 0.05, and indicates that performance is different between the PL dual-task condition, the standalone condition and the VSSP dual-task condition, we will determine if the PL (but not the VSSP) is involved in the translation between Arabic digits and non-symbolic quantities if *t*‐test is significant at *p* < 0.05, and indicates that performance is different between the PL dual-task condition and the standalone condition but the *t*‐test indicates that there is no significant difference between the PL dual-task condition and the VSSP dual-task condition then we will conclude that there is an additional WM load from completing two tasks simultaneously; however, we cannot be specific about the component of WM involved secondary tasks if performance in the secondary task is significantly different between the PL dual-task condition than in the PL standalone condition, we will conclude that the PL is involved in translating between Arabic digits and non-symbolic quantities if performance in the secondary task is significantly different between the VSSP dual-task condition and the VSSP standalone condition, we will look at the means to determine if the VSSP is involved in translating between Arabic digits and non-symbolic quantities	if we observe PL involvement in either the primary or secondary task analysis we will conclude that participants use verbal labels in translating between Arabic digits and non-symbolic quantities if we observe VSSP involvement in either the primary or secondary task analysis, we will conclude that participants use visual strategies in translating between Arabic digits and non-symbolic quantities if we observe both VSSP and PL involvement, we will conclude that WM is required in translating between Arabic digits and non-symbolic quantities; however, we cannot be specific about which component if we observe no WM involvement (either PL or VSSP), we will conclude translating between Arabic digits and non-symbolic quantities may be automatic
**RQ 3a**. Does processing of Arabic digits differ for small and large quantities?	in the **symbolic comparison** condition, we expect no difference in the processing of small and large quantities. For both small and large quantities, we expect to see PL involvement. This means we will expect to see a difference in the **symbolic primary task** performance between the PL dual-task condition and the standalone condition, for both small and large quantities	based on the effect size calculations described above, the smallest effect size of interest for this RQ is 0.5. Using a power level of 90%, an alpha level of 0.05 and a minimum effect size of Cohen’s *d* = 0.5, the maximum sample required for this RQ is 36	for small quantities paired samples *t*‐test (standalone symbolic comparison versus symbolic comparison with PL dual-task) for large quantities paired samples *t*‐test (standalone symbolic comparison versus symbolic comparison with PL dual-task)	the calculated effect sizes represent either: a reduction in primary accuracy of 5 trials or a reduction in secondary accuracy of 2 trials	if *t*-tests for both small and large quantities are significant (*p* < 0.05), we will conclude that processing both small and large Arabic digits involves the PL	if *t*-tests for either small or large quantities are not significant, we will conclude that processing of these quantities may be automatic
**RQ 3b**. Does processing of non-symbolic representations differ for small and large quantities?	in the **non-symbolic comparison** condition, we expect that small quantities will be processed automatically, while large quantities will involve the VSSP this means we will expect to see a difference in the **non-symbolic primary task** performance between the VSSP dual-task condition and the standalone condition for large quantities, but no decrease in performance for small quantities	using a power level of 90%, an alpha level of 0.05 and a minimum effect size of Cohen’s *d* = 0.35, the maximum sample required for this RQ is 72	for small quantities paired samples *t*‐test (standalone non-symbolic comparison versus non-symbolic comparison with VSSP dual-task) for large quantities paired samples *t*‐test (standalone non-symbolic comparison versus non-symbolic comparison with VSSP dual-task)	the calculated effect sizes represent either: a reduction in primary accuracy of 5 trials, or a reduction in secondary accuracy of 2 trials	if *t*-tests for both small and large quantities are significant (*p* < 0.05), we will conclude that processing both small and large Arabic digits involves the VSSP	if *t*-tests for either small or large quantities are not significant, we will conclude that processing of these quantities may be automatic
**RQ 3c**. Does translation between Arabic and non-symbolic representations differ for small and large quantities?	in the **cross-modal comparison** condition, we expect no difference in the processing of small and large quantities. For both small and large quantities, we expect to see PL involvement this means we will expect to see a difference in **cross-modal primary task** performance between the PL dual-task condition and the standalone condition, for both small and large quantities	using a power level of 90%, an alpha level of 0.05 and a minimum effect size of Cohen’s *d* = 0.33, the maximum sample required for this RQ is 81	for small quantities paired samples *t*‐test (standalone cross-modal comparison versus cross-modal comparison with PL dual-task) for large quantities paired samples *t*‐test (standalone cross-modal comparison versus cross-modal comparison with PL dual-task)	the calculated effect sizes represent either: a reduction in primary accuracy of 5 trials or a reduction in secondary accuracy of 2 trials	if *t*-tests for both small and large quantities are significant (*p* < 0.05), we will conclude that translating between Arabic digits and non-symbolic quantities involves the PL for both small and large quantities	if *t*-tests for either small or large quantities are not significant, we will conclude that translating between these quantities is automatic

To answer our three research questions, we will conduct all of the analyses described in table 4 (analysis plan column). The alternative interpretations of the different potential outcomes are provided below. For all analyses described below, a ‘decrease in performance’ refers to either a decrease in accuracy or an increase in reaction times between the stated conditions.

## Appendix D

### Exploratory analyses

**Table 5.** Exploring potential speed–accuracy trade-offs across the symbolic conditions.

correlation accuracy–RT	*r*	*p*
symbolic standalone	0.2	0.07
symbolic PL	0.3	0.009
symbolic VSSP	0.13	0.26

RQ3 regarded the possible differential impact of interference for small and large quantities and numbers. In this case, our pre-registered analyses were dependent on our predictions regarding which WM component would be involved with each type of primary task (RQs 1−2). So, for symbolic and cross-modal comparison, we had hypothesized PL involvement and, therefore, only pre-registered investigating the impact of interference separately for small and large-number trials for PL interference. Similarly, for non-symbolic comparison, we had only hypothesized VSSP WM involvement. Our results, however, were more nuanced. To present the complete picture of the effects of the interference conditions, in figure 5, we also report the results of paired sample *t*-tests for comparisons of with and without interference separately for small- and large-number trials in the interference conditions that were not originally expected (i.e. in the case of symbolic and cross-modal comparison for PL and in the case of non-symbolic comparison for VSSP). These are presented with grey arrows in figure 5. These results should be interpreted with caution given their exploratory nature.

Further inspection of the overall results in figure 5, especially in the case of the symbolic comparison primary task, one may wonder whether our findings were affected by a speed–accuracy trade-off. Specifically, we found that, under both interference conditions in the symbolic task, participants were more accurate but slower compared to the standalone condition. Initially, we had hypothesized that significant increases in RT would be reflective of interference effects, but in this case, rather than slowing because of the interference, this RT effect may have resulted from improved attention to the primary task under interference and being more accurate. To test this assumption, we ran the respective correlations (table 5). We found a significant but small correlation between accuracy and RT only in the case of PL interference but this correlation did not significantly differ from the respective symbolic standalone and symbolic VSSP speed–accuracy *r* values, indicating that there was no significant change in a speed–accuracy trade-off across these conditions (standalone versus PL interference: *z* = −0.78, *p* = 0.43; standalone versus VSSP interference: *z* = 0.49, *p =* 0.62; PL interference versus VSSP interference: *z* = −1.76, *p* = 0.08).

Finally, it is well known that adults perform best in symbolic, then non-symbolic and finally cross-modal comparison tasks [52]. We checked and confirmed that this was the case with our accuracy data too, *F*_2,240_ = 26.47, *p* < 0.001 (*M*_Symbolic standalone_ = 0.96, s.d. = 0.03; *M*_Non-symbolic standalone_ = 0.93, s.d. = 0.06; *M*_Cross-modal standalone_ = 0.91, s.d. = 0.06; *post hoc* comparisons: symbolic versus non-symbolic: *t* = 4.01, *p* < 0.001; non-symbolic versus cross-modal: *t* = 3.25, *p* = 0.001; symbolic versus cross-modal: *t* = 7.26, *p* < 0.001).

^1^
This minor change from the Stage 1 manuscript was approved by PCI RR on 16 March 2023.
